# Structure-Activity Relationship (SAR) and *in vitro* Predictions of Mutagenic and Carcinogenic Activities of Ixodicidal Ethyl-Carbamates

**DOI:** 10.1155/2020/2981681

**Published:** 2020-11-21

**Authors:** María G. Prado-Ochoa, Maribel Strassburger-Madrigal, Rafael Camacho-Carranza, Jesús J. Espinosa-Aguirre, Ana M. Velázquez-Sánchez, Victor H. Vázquez-Valadez, Enrique Angeles, Fernando Alba-Hurtado, Marco A. Muñoz-Guzmán

**Affiliations:** ^1^Departamento de Ciencias Biológicas, Facultad de Estudios Superiores Cuautitlán, Universidad Nacional Autónoma de México, Mexico; ^2^Programa de Maestría en Ciencias de la Producción y de la Salud Animal, Universidad Nacional Autónoma de México, Mexico; ^3^Departamento de Medicina Genómica y Toxicología Ambiental, Instituto de Investigaciones Biomédicas, Universidad Nacional Autónoma de México, Mexico; ^4^Laboratorio de Química Medicinal, Departamento de Ciencias Químicas, Facultad de Estudios Superiores Cuautitlán, Universidad Nacional Autónoma de México, Mexico

## Abstract

Ethyl-4-bromophenyl-carbamate (LQM 919) and Ethyl-4-chlorophenyl-carbamate (LQM 996) are compounds that inhibit egg-laying and hatching of tick larvae that are resistant to conventional ixodicides. The structure-activity relationship (SAR) to get the endpoint predictions of mutagenicity and carcinogenicity of the LQM 919 and LQM 996 was performed and the absence of mutagenicity was confirmed by Ames test. SAR analysis show no structural alerts indicating the ability of ethyl-carbamates to bind biomolecules or estrogen receptors. Endpoint of mutagenicity with and without metabolic activation showed that the ethyl-carbamates were negative (p <0.05) for mutagenicity induction in strains TA97, TA98, TA102, TA1535, TA1537 and TA1538 of *Salmonella typhimurium*. Pre-incubation with different ethyl-carbamate concentrations did not increase the number of spontaneously reverting colonies; moreover, the compounds did not induce a concentration-dependent increase in the number of reverting colonies in any of the strains used. This confirmed the absence of mutagenic activity in this test system. Exogenous metabolic activation did not modify these observations; suggesting that no metabolites with mutagenic activity were present. The endpoint of carcinogenicity in rats were negative for LQM 919 (p <0.05,) and LQM 996 (p <0.001). The results of the present study strongly suggest that ethyl-carbamates do not represent a risk for cancer in mammals.

## 1. Introduction

Currently, regulation of chemical substances that are used daily as pesticides (i.e. ixodicides) is of great relevance globally. The European Chemicals Agency (ECHA), the Organization for Economic Cooperation and Development (OECD), and the Environmental Protection Agency (EPA), are the most important organizations responsible for setting the guidelines for regulation of chemical substances.

These organizations generally accept *in silico* methods based on computational modeling of chemical structure and biological activity of a compound to predict its toxicity without the use of biological tests. This allows reducing costs, the number of experimental animals, risks in handling hazardous chemicals, and the time used to perform *in vitro* or *in vivo* tests [1].

*Rhipicephalus microplus*, considered the most important parasitic tick on cattle in tropical and subtropical areas worldwide, represents a great threat to the livestock industry due to the diseases it transmits as a vector and the direct economic losses it causes [2]. Excessive use of chemical substances for the control of ticks in cattle (ixodicides) has resulted in development of tick strains that are resistant to the majority of commercial ixodicides in several regions of the world [3]. This has generated the need to develop new molecules to which no tick strains are currently resistant. We have shown that ethyl-4-bromophenyl-carbamate (LQM 919) and ethyl-4-chlorphenyl-carbamate (LQM 996) inhibit egg-laying and hatching of *Rhipicephalus microplus* larvae and this inhibition was observed both in strains that are resistant to other ixodicides and in those that are susceptible to them [4, 5]. These carbamates alter the reproductive organs, vitellogenesis, and viability of ovarian cells in *R. microplus* and cause harm that is suggestive of apoptosis [6]. These effects were found to be independent of acetylcholinesterase inhibition [7].

The toxicity of LQM 919 and LQM 996 must be evaluated before these compounds are proposed for the safe control of ticks. Following the criteria of the OECD, these carbamates are found to have low acute and subchronic toxicity in rats [8, 9]. Nevertheless, these compounds produce an increase in micronucleated reticulocytes in the peripheral blood of rats as well as alterations in the cell cycle of human lymphocytes suggesting that these compounds cause DNA damage [10]. Other carbamates such as vinyl-carbamate and ethyl-carbamate (urethane) are carcinogenic and produce tumors in several organs of rodents [11, 12]. This suggests a potential risk for the application of carbamates in animals used for human food and to the persons who apply these products. Therefore, this study aimed to evaluate the mutagenic and carcinogenic potential of the ethyl-carbamates LQM 919 and LQM 996 *in silico*, as well as to corroborate their mutagenic capacity using the Ames test.

## 2. Materials and Methods

### 2.1. Carbamates

The ethyl-carbamates used in this study were designed, synthesized, and patented at the National Autonomous University of Mexico using a benzimidazole molecule as the structural base, as described by Angeles et al. [13]. The chemical structures, nomenclature, molecular weights, simplified molecular input line entry system (SMILES), octanol/water partition coefficient (Kow) and identification codes of ethyl-carbamates are shown in Table 1.

### 2.2. *In silico* Studies

Structure-activity relationship (SAR) for endpoint predictions of mutagenicity and carcinogenicity of the ethyl-carbamates LQM 919 and LQM 996 was performed by Read-across-analysis using the QSAR Toolbox software according to workflow of the hazard assessment process: Input; Profiling; Endpoint data collection; category definition; data gap filling, and finally, reporting [14]. The partition coefficients (Log Kw) of each carbamate were used as descriptors. A conformational study of LQM 919 and LQM 996 was conducted; the molecules were modeled using the Toolbox modeler tool and were double checked by comparing each SMILES code. Profiling was carried out using the OECD HPV chemical categories, substance type, and US-EPA New Chemical Categories profiling methods [15, 16].

The carbamate structures were optimized by PM3 (Molecular Orbital Model) and were categorized using the DICE (Dynamic Integrated model of Climate and the Economy) model to obtain the DICE coefficient [17, 18]. Molecular feature parameters (atom centered, fragments and cycles) and atomic characteristics (count H attach, hybridization and cyclic) were used to obtain a set of chemical analogues with greater than 50% similarity to the carbamates LQM 919 and LQM 996. The endpoint predictions of mutagenicity on strains TA97, TA98, TA102, TA1535, TA1537, and TA1538 of *Salmonella typhimurium* with and without simulation of S9 metabolic activation, and endpoint predictions of carcinogenicity on rats by the oral route of ethyl-carbamates were determined. The endpoint value predictions were based on five neighbors' values. Prediction confidence was established and categorized as moderate (p <0.05), strong (p <0.01), or very strong (p <0.001).

### 2.3. Ames' *in vitro* Assay

*S. typhimurium* strains TA98 (his D3052, rfa National Autonomous University of Mexico, uvrB, bio-, pKM101[ApR]), TA100 (his G46, rfa, uvrB, bio-, pKM101[ApR]), TA1535 (his G46, rfa, uvrB, bio-), TA1537 (his C3076, rfa, uvrB, bio-), and TA102 (His G428, rfa, bio-, pKM101[ApR], pAQ1[TtR]) were used; these were maintained at the Mutagens and Carcinogens Laboratory of the Biomedical Research Institute, National Autonomous University of Mexico. Before the assays were carried out, single colonies were isolated from each strain, and their genotypic characteristics were confirmed using the procedure described by Maron and Ames [19]. The toxicity of varying concentrations of the studied compounds was assessed by microscopic observation of bacterial growth in the selected dishes. Homogeneous distribution of bacteria was considered indicative of no toxicity, whereas loss of homogeneity and the dose-dependent decrease in reverting colonies was considered indicative of toxicity.

Extract S9 from the livers of Sprague-Dawley male rats, induced with phenobarbital and B-naphthoflavone, was prepared and stored (-80°C) according to the modified method described by Escobar-García et al. [20].

One colony from each strain grown in NB solid medium was transferred to 10 mL of NB-Oxoid broth and kept under constant stirring (150 rpm) for 12 h. Subsequently, 0.1 mL of cultures from each strain (approximately 10^8^ cells) was pre-incubated for 30 min with a mixture of 0.5 mL of S9 mix or 0.5 mL of phosphate buffer at pH 7.4 and varying final concentrations of the studied carbamates (0, 0.1, 0.2, 0.4, 0.5 and 1 mg/mL) that had been previously diluted with dimethyl sulfoxide (DMSO). The lowest concentration (0.1 mg/mL) was selected considering the results of previous assays that demonstrated no toxicity, and the concentration was increased for the remaining assays.

The pre-incubated mix was homogenized into 2 mL of molten top agar (0.6% agar, 0.5% NaCl, 0.5 mM biotin, and 0.05 mM L-histidine) and subsequently distributed homogeneously on the surface of minimal agar plates. The plates were incubated at 37°C for 48 h, and the number of reverting colonies in each plate was counted. The criteria for considering carbamates as mutagenic were (1) a dose-dependent increase in the number of reverting colonies or (2) the number of induced reverting colonies greater than or equal to double the amount of reverting colonies found in the negative (0 mg/mL) control plates [21, 22].

Positive controls (known specific mutagens) and negative controls were included in each assay. The specific positive controls were 2-nitrofluorene (10 *μ*g/plate for TA98 without S9), sodium azide (1 *μ*g/plate for TA100 and TA1535 without S9), picrolonic acid (100 *μ*g/plate for TA1537 without S9), 4-Nitroquinoline-N-oxide (1 *μ*g/plate for TA102 without S9), and 2-aminoanthracene (2 *μ*g/plate for all strains with S9). All the mutagens were previously diluted in DMSO except for sodium azide, which was dissolved in water. The negative control for all strains was phosphate buffer at pH 7.4 with 2% DMSO [23].

## 3. Results

### 3.1. Profiling

Table 2 shows the profiling results of LQM 919 and LQM 996. The qualitative analysis did not show structural alerts that indicated the ability of these ethyl-carbamates to either produce damage or bind to biomolecules.

### 3.2. Mutagenicity Predictions

Table 3 shows the endpoint predictions of mutagenicity in six strains of *S. typhimurium*. The ethyl-carbamates studied were negative (p <0.05) for the mutagenicity endpoint in all strains studied with and without simulation of metabolic activation using S9.

### 3.3. Carcinogenicity Predictions

**Figure 1:**
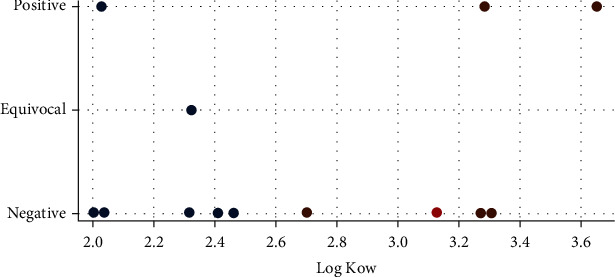
Read across prediction of summary carcinogenicity from LQM 919. The red dot represents the predicted value for target chemical (LQM 919), brown dots represent the observed value for the target neighbours (analogues) used for read-across and blue dots represent the experimental results available for the analogues but not used for read-across. Predicted LQM 919 value: “Negative”, p <0.001.

The read across predictions of carcinogenicity in rats were negative for LQM 919 (p <0.05, moderate confidence) as well as for LQM 996 (p <0.001, strong confidence).  Figure 1 shows the summary carcinogenicity predictions from LQM 919 and neighbours (analogues) used for read-across analysis (a similar result was observed for the LQM 996).

### 3.4. *In vitro* Ames' Assay

Tables 4 and 5 show the average number of reverting colonies in strains exposed to different LQM 919 and LQM 996 concentrations with and without S9 metabolic activation, respectively. The number of spontaneous reverting colonies at all the concentrations used for both carbamates did not show a concentration-dependent increase; moreover, this number was not greater than or equal to double the number of spontaneous reverting colonies of each strain in the respective negative control. In general, greater concentrations of these ethyl-carbamates resulted in thinning out of background growth.

## 4. Discussion

The genotoxicity induced by pesticides is considered one of the most severe undesirable effects in mammals. When a pesticide reacts with nuclear DNA, it is considered potentially mutagenic and carcinogenic to the exposed organisms; therefore, its use in therapeutics may be limited. For the aforementioned reasons, when the use of new pesticides is proposed, their potential as a possible mutagen and carcinogen should be evaluated before being widely used in the environment. In the present study, *in silico* predictive analyses and studies on *in vitro* effects showed that the evaluated ethyl-carbamates do not present a mutagenic or carcinogenic risk.

Most mutagens and carcinogens or their metabolites covalently bind to cellular macromolecules such as DNA and proteins before producing changes in their structure, sequence, or function [24]. Some ethyl-carbamates such as urethane reportedly bind to liver cell DNA before producing tumors in mouse liver or to lung cell DNA before producing tumors in rat lungs [25, 26]. In this study, read-across-analysis revealed no DNA-binding or protein-binding alerts in the molecular structure of LQM 919 and LQM 996. These results suggest that these ethyl-carbamates have a low probability of affecting DNA replicative processes that could alter its sequence.

High levels of estrogen have been associated with tumor production both in experimental models and in epidemiological studies in humans [27–29]. One of the mechanisms of estrogen carcinogenicity is its binding to the specific nuclear receptor ER-*α*, exerting a potent stimulus on cell proliferation through increased production of growth factors [30]. Some pesticides such as Acetochlor, Dicofol, Endosulfan, Propoxur, and Lindane mimic estrogen activity in terms of structure and function (xenoestrogens) as these can bind estrogen receptors and stimulate tumor production [31, 32]. *In silico* analysis did not show structural alerts that indicate probable estrogen receptor binding in the ethyl-carbamates studied; therefore, the risk that they can induce cancer by this mechanism is low. In addition to the above, the read across prediction of carcinogenicity in rats was negative for both ethyl-carbamates; therefore, their chemical structure does not seem to present a risk of cancer induction either. However, other alerts indicated possible toxicity. According to the Cramer classification (estimation of the threshold of toxicological concern) these compounds were categorized within class III, which includes compounds with a chemical structure that does not allow an initial impression of safety, and suggests significant toxicity. The US-EPA New Chemical Categories prediction indicated that these are neutral organic compounds related to probable environmental toxicity for some aquatic species [24].

The Ames' assay is used worldwide to determine the mutagenic capability of pesticides [33, 34]. This test is highly sensitive, and depending on the strain of *S. typhimurium* used, it can reveal different types of mutations. Strains TA98, TA1535, and TA1538 are sensitive to frameshift mutations, strains TA97, TA100, and TA1535 are sensitive to C/G base pair exchanges, and strain TA102 is sensitive to A/T base pair exchanges and mutations caused by oxidative damage [22, 35]. In this study, the results of read-across analysis predicted that the evaluated ethyl-carbamates have no mutagenic potential in any of the six strains of *S. typhimurium* studied. These predictions were consistent with the results obtained in the Ames *in vitro* assay, and in both cases, metabolic activation of ethyl-carbamates was considered. Some carbamates such as Propoxur, Carbaryl, and Methomyl acquire mutagenic activity when subjected to exogenous metabolic activation [36, 37]. We did not observe differences in spontaneous reversion induced by the ethyl-carbamates under study with or without exogenous metabolic activation. The *in silico* and *in vitro* results obtained in this study indicated the absence of mutagenic activity in the ethyl-carbamates or their metabolites.

In previous studies, we observed that the ethyl-carbamates used in this study induce an increase in micronucleated reticulocytes in rats, which could be the result of DNA damage [10]. Furthermore, we observed an increase in thiobarbituric acid reactive species (oxidative stress) that can cause damage to macromolecules including DNA [8]. However, the results of the present study suggest that these ethyl-carbamates did not represent a risk for cancer in mammals.

## 5. Conclusions

*In silico* studies predicted that the ethyl-carbamates LQM 919 and LQM 996 are negative for mutagenicity and carcinogenicity. The *in vitro* Ames test confirmed the absence of mutagenic capacity in these compounds.

## Figures and Tables

**Table 1:** Nomenclature, chemical structure and characteristics of ethyl-carbamates.

	LQM 919 Ethyl (4-bromophenyl) carbamate	LQM 996 Ethyl (4-chlorophenyl) carbamate
Chemical structure	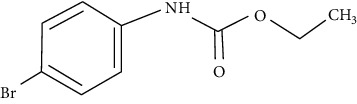	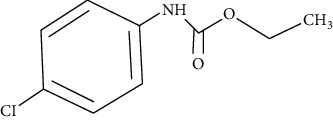
Molecular weight	244	199.63
SMILES	CCOC(=O)Nc1ccc(Br)cc1	CCOC(=O)Nc1ccc(cl)cc1
Descriptors	Log Kow 3.13	Log Kow 2.89

**Table 2:** Profiling results of Structure-Activity Relationship (SAR) analysis of ixodicidal ethyl-carbamates.

Chemical or biological mechanism	LQM 919	LQM 996
DNA binding by OECD	No alert found	No alert found
Estrogen receptor binding	Non binder, without OH or NH2 group	Non binder, without OH or NH2 group
Protein binding by OECD	No alert found	No alert found
Superfragments	No superfragment	No superfragment
Toxic hazard classification by Cramer (original)	High (class III)	High (class III)
US-EPA new chemical categories	Neutral organics	Neutral organics

**Table 3:** Predictions of mutagenicity of ethyl-carbamates on different strains of *Salmonella typhimurium* with and without S9 metabolic activation.

CARBAMATE	Strain	With S9 activation	P-value	Without S9 activation	P-value
LQM 919	TA 97	Negative	p <0.001∗∗∗	Negative	p =0.100
	TA 98	Negative	p <0.001∗∗∗	Negative	p <0.05∗
	TA 102	Negative	p <0.01∗∗	Negative	p <0.01∗∗
	TA 1535	Negative	p <0.001∗∗∗	Negative	p <0.01∗∗
	TA 1537	Negative	p <0.001∗∗∗	Negative	p <0.05∗
	TA 1538	Negative	p <0.001∗∗∗	Negative	p <0.001∗∗∗
LQM 996	TA 97	Negative	p <0.001∗∗∗	Negative	p <0.001∗∗∗
	TA 98	Negative	p <0.001∗∗∗	Negative	p <0.01∗∗
	TA 102	Negative	p <0.01∗∗	Negative	p <0.001∗∗∗
	TA 1535	Negative	p <0.001∗∗∗	Negative	p <0.01∗∗
	TA 1537	Negative	p <0.001∗∗∗	Negative	p <0.01∗∗
	TA 1538	Negative	p <0.01∗∗	Negative	p <0.001∗∗∗

∗ moderate confidence; ∗∗ strong confidence; ∗∗∗ very strong confidence.

**Table 4:** Mutagenicity of carbamate LQM 919 towards *Salmonella typhimurium* TA98, TA100, TA1535, TA1537 and TA102 strain with or without S9 metabolic activation.

	No. of *his*∗ revertant colonies/plate (mean ± SD)									
Concentration (mg/mL)	TA98		TA100		TA1535		TA1537		TA102	
	-S9	+S9	-S9	+S9	-S9	+S9	-S9	+S9	-S9	+S9
0 (-)	18.5 ± 3.2	27.7 ± 2.5	104.1 ± 10.8	120 ± 9	9.1 ± 3.9	16.5 ± 1.9	9.1 ± 3	14.6 ± 3	319.5 ± 75.7	330 ± 66
0.1	16.3 ± 4.9	31 ± 6.2	112 ± 20.7	133.3 ± 10.5	10 ± 1.7	13.6 ± 1.5	10.6 ± 2.5	14 ± 5.2	225 ± 26.5	347.6 ± 41.5
0.2	20.3 ± 4.1	28.7 ± 7.1	107.6 ± 15.8	118.6 ± 4.5	6 ± 3.4	9.3 ± 2.0	4.3 ± 0.5	9.6 ± 2	219.6 ± 5.8	265 ± 13
0.4	16.3 ± 2	30 ± 7.5	125.6 ± 19.4	89.6 ± 19.8	11 ± 2.6	11 ± 4.3	6.33 ± 3.2	9.3 ± 2	118.3 ± 21.9	250.3 ± 15.8
0.5	20.3 ± 4.5	32.3 ± 8.6	78 ± 2	106.3 ± 16.8	4 ± 3.4	12 ± 3	4 ± 2.6	9.6 ± 1.5	80.6 ± 2.8	101.6 ± 89.2
1	10 ± 5.2	25.6 ± 5.1	35 ± 22.9	28.3 ± 19.4	5 ± 1	6.6 ± 1.5	2 ± 2	6 ± 4.5	3.3 ± 5.7	0
Limit value:	37	55.4	208.2	240	18.2	33	18.2	29.2	639	660
Positive controls:										
2-NF	2017 ± 460									
Sodium azide			1666 ± 99		731 ± 77.2					
Picrolonic acid							48.33 ± 25.1			
4-NQO									1573 ± 61	
2-AA		1831 ± 163		2277 ± 146		765 ± 45		441 ± 99		2050 ± 93

S9 = Microsomal fraction of liver of rats treated with phenobarbital and beta-naphthoflavone enriched with NADP and glucose-6-P; 2-NF =2-nitrofluorene; 4-NQO =4-nitroquinolido N-oxide; 2-AA =2-aminoanthracene.

**Table 5:** Mutagenicity of carbamate LQM 996 towards *Salmonella typhimurium* TA98, TA100, TA1535, TA1537 and TA102 strain with or without S9 metabolic activation.

	No. of *his*∗ revertant colonies/plate (mean ± SD)									
Concentration (mg/mL)	TA98		TA100		TA1535		TA1537		TA102	
	-S9	+S9	-S9	+S9	-S9	+S9	-S9	+S9	-S9	+S9
0 (-)	18.5 ± 3.2	27.7 ± 2.5	104.1 ± 10.8	120 ± 9	9.1 ± 3.9	16.5 ± 1.9	9.1 ± 3	14.6 ± 3	319.5 ± 75.7	330 ± 66
0.1	20.3 ± 4.1	28.3 ± 6.1	94.6 ± 27.7	92.6 ± 5.5	8.3 ± 3.2	7.6 ± 4.6	7.3 ± 3.2	8.3 ± 1.1	395.3 ± 6.1	473 ± 17.5
0.2	20 ± 5	30.3 ± 4.0	96 ± 3	101.3 ± 12.3	4 ± 1.7	10.6 ± 3.5	4.6 ± 2	8.3 ± 2.5	251.6 ± 49.8	419.6 ± 31.6
0.4	17 ± 4	22.6 ± 8	83.6 ± 17.6	95 ± 4.3	2.6 ± 1.5	11.6 ± 1.5	1.6 ± 2.8	5.3 ± 3	241.3 ± 49.1	367.3 ± 58.2
0.5	18.3 ± 4	30.3 ± 1.1	96.6 ± 12.5	81.6 ± 16.6	2.6 ± 2.3	4.6 ± 3.2	0	0	194.3 ± 43	335.6 ± 18
1	13 ± 1.7	21.3 ± 3	34.6 ± 26.2	0	0	6.6 ± 2.8	0	0	150 ± 16.7	185.3 ± 29.2
Limit value:	37	55.4	208.2	240	18.2	33	18.2	29.2	639	660
Positive controls:										
2-NF	2017 ± 460									
Sodium azide			1666 ± 99		731 ± 77.2					
Picrolonic acid							48.33 ± 25.1			
4-NQO									1573 ± 61	
2-AA		1831 ± 163		2277 ± 146		765 ± 45		441 ± 99		2050 ± 93

S9 = Microsomal fraction of liver of rats treated with phenobarbital and beta-naphthoflavone enriched with NADP and glucose-6-P; 2-NF =2-nitrofluorene; 4-NQO =4-nitroquinolido N-oxide; 2-AA =2-aminoanthracene.

## Data Availability

The data used to support the findings of this study are available from the corresponding author upon request.
